# Endoplasmic reticulum stress enhances the expression of TLR3-induced TSLP by airway epithelium

**DOI:** 10.1152/ajplung.00378.2023

**Published:** 2024-03-12

**Authors:** Prabuddha S. Pathinayake, Alan C.-Y. Hsu, Kristy S. Nichol, Jay C. Horvat, Philip M. Hansbro, Peter A. B. Wark

**Affiliations:** ^1^Immune Health Program, Hunter Medical Research Institute and School of Medicine and Public Health, University of Newcastle, Newcastle, New South Wales, Australia; ^2^Immune Health Program, Hunter Medical Research Institute and School of Biomedical Sciences and Pharmacy, University of Newcastle, Newcastle, New South Wales, Australia; ^3^Department of Respiratory and Sleep Medicine, John Hunter Hospital, Newcastle, New South Wales, Australia; ^4^Faculty of Science, School of Life Sciences, Centre for Inflammation, Centenary Institute, University of Technology Sydney, Sydney, New South Wales, Australia; ^5^School of Medicine, Monash University, Melbourne, Victoria, Australia; ^6^AIRMED Alfred Health, Melbourne, Victoria, Australia; ^7^Signature Research Program in Emerging Infectious Diseases, Duke-National University of Singapore (NUS) Graduate Medical School, Singapore, Singapore

**Keywords:** ER stress, severe neutrophilic asthma, TSLP, unfolded protein response

## Abstract

Thymic stromal lymphopoietin (TSLP) is an epithelial-derived pleiotropic cytokine that regulates T-helper 2 (Th2) immune responses in the lung and plays a major role in severe uncontrolled asthma. Emerging evidence suggests a role for endoplasmic reticulum (ER) stress in the pathogenesis of asthma. In this study, we determined if ER stress and the unfolded protein response (UPR) signaling are involved in TSLP induction in the airway epithelium. For this, we treated human bronchial epithelial basal cells and differentiated primary bronchial epithelial cells with ER stress inducers and the TSLP mRNA and protein expression was determined. A series of siRNA gene knockdown experiments were conducted to determine the ER stress-induced TSLP signaling pathways. cDNA collected from asthmatic bronchial biopsies was used to determine the gene correlation between ER stress and TSLP. Our results show that ER stress signaling induces TSLP mRNA expression via the PERK-C/EBP homologous protein (CHOP) signaling pathway. AP-1 transcription factor is important in regulating this ER stress-induced TSLP mRNA induction, though ER stress alone cannot induce TSLP protein production. However, ER stress significantly enhances TLR3-induced TSLP protein secretion in the airway epithelium. TSLP and ER stress (PERK) mRNA expression positively correlates in bronchial biopsies from participants with asthma, particularly in neutrophilic asthma. In conclusion, these results suggest that ER stress primes TSLP that is then enhanced further upon TLR3 activation, which may induce severe asthma exacerbations. Targeting ER stress using pharmacological interventions may provide novel therapeutics for severe uncontrolled asthma.

**NEW & NOTEWORTHY** TSLP is an epithelial-derived cytokine and a key regulator in the pathogenesis of severe uncontrolled asthma. We demonstrate a novel mechanism by which endoplasmic reticulum stress signaling upregulates airway epithelial TSLP mRNA expression via the PERK-CHOP signaling pathway and enhances TLR3-mediated TSLP protein secretion.

## Graphical abstract

**Figure:**
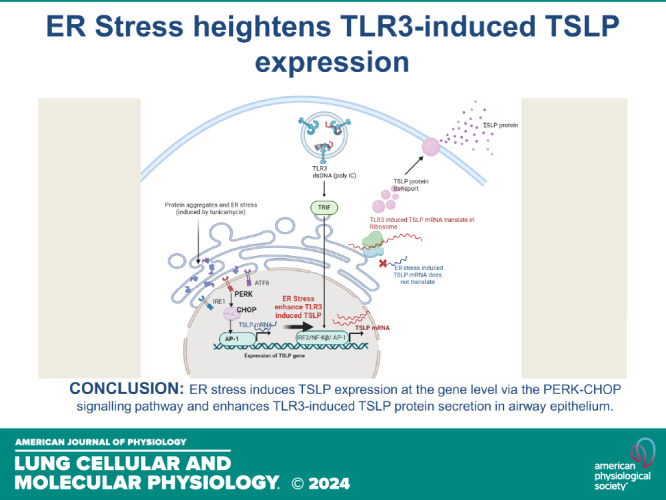

## INTRODUCTION

Thymic stromal lymphopoietin (TSLP) is an innate IL-7-like cytokine that is predominantly expressed in the epithelium of the lung, gut, and skin (1), though it is also expressed by mast cells, airway smooth muscle cells, fibroblasts, dendritic cells, trophoblasts, and neoplastic cells (2). TSLP strongly activates antigen-presenting dendritic cells (DCs) and promotes the differentiation of naive human CD4^+^ T cells to T-helper type-2 (Th2) cells (3–5). It also regulates DC-mediated CD4^+^ T-cell homeostasis by expanding autologous CD4^+^ T-cell populations (6). Moreover, TSLP suppresses the activity of regulatory T cells (Tregs) in the lungs. It directly and selectively reduces IL-10 production by Treg in both healthy and asthmatic patients but more potently in asthmatics (7). It also suppresses antigen-specific Tregs (8) suggesting a role for TSLP in suppressing allergen tolerance in the airways. In this context, it is increasingly thought to play an important role in promoting airway inflammation in asthma. Increased expression of TSLP has been found in bronchial mucosa and bronchoalveolar lavage fluid (BALF) of severe asthma patients (9). The TSLP concentration in BALF of those patients was correlated with poor lung function and the dose of corticosteroids used, suggesting that this cytokine is a potential biomarker of severe uncontrolled asthma (10).

Various stimulants such as TLR ligands, Nod2 ligands, viral, bacterial and fungal infections, allergens, helminths, environmental pollutants such as diesel exhaust, and cigarette smoke can stimulate TSLP secretion in the lungs (2). In DCs, TSLP secretion induced by *Candida albicans* and β-glucans is enhanced by signals emanating from the endoplasmic reticulum (ER) stress response, specifically the unfolded protein response (UPR) sensors, inositol-requiring transmembrane kinase/endonuclease (IRE)-1, and protein kinase R-like ER kinase (PERK). However, ER stress alone was not sufficient to induce TSLP (11). The UPR is an adaptive response when cells undergo ER stress due to physiological demand or aberrations in protein folding in the ER (12). The UPR activates a complex signaling cascade that involves the activation of various transcription factors. C/EBP homologous protein (CHOP) is a multifunctional transcription factor that activates the UPR and induces apoptosis and inflammatory responses in cells (13). CHOP is involved in IL-1β production in macrophages (14) and IL-23 in DCs (15) and also plays an important role as a gene transcription enhancer or inhibitor. It can form a heterodimer with C/EBP family transcription factors and activate the expression of certain activator protein (AP)-1-targeted genes (16). It can also inhibit certain genes by dimerizing with C/EBP and liver-enriched transcriptional activator protein (LAP) (17).

Here, we demonstrate a novel mechanism of TSLP mRNA induction in the airway epithelium via ER stress/UPR-induced CHOP signaling. We show that ER stress also enhances TSLP protein secretion induced by TLR3 agonists such as polyinosinic:polycytidylic acid (poly I:C) in the airway epithelium. Furthermore, we provide evidence for an association between ER stress and TSLP expression in clinical bronchial biopsies from severe asthma patients, in particular those with neutrophilic inflammation. These data define a novel mechanism underpinning increased TSLP expression in the asthmatic epithelium in severe asthma and during virus-induced acute asthma.

## MATERIALS AND METHODS

### Cell Culture

#### BEGM media.

To prepare BEGM complete media, bronchial epithelial cell (BEC) growth medium (BEGM; Lonza) supplemented with bovine pituitary extract (BPE), insulin, gentamicin sulfate (GA-1000), retinoic acid, transferrin, tri-iodothyronine (T3), epinephrine, rhEGF, hydrocortisone, penicillin/streptomycin (2%), and amphotericin B (fungizone, 1%). To prepare BEGM minimal media, penicillin/streptomycin (2%), amphotericin B (fungizone, 1%), insulin-transferrin-sodium (ITS, 1%; Sigma), and linoleic acid-albumin from bovine serum albumin (0.5%; Sigma) were added into bronchial epithelial basal medium (BEBM).

#### Air-liquid interface media.

Air-liquid interface (ALI) initial media comprised 50% BEBM-50% DMEM containing hydrocortisone, bovine insulin, epinephrine, transferrin (all 0.1%), bovine pituitary extract (0.4%) (all from Lonza) and ethanolamine (final concentration 80 μM), MgCl_2_ (final concentration 0.3 mM), MgSO_4_ (final concentration 0.4 mM), bovine serum albumin (final concentration 0.5 mg/mL), amphotericin B (final concentration 250 μg/mL), all-trans retinoic acid (30 ng/mL), and penicillin/streptomycin (2%) with rhEGF (10 ng/mL). For ALI final media, recombinant human epidermal growth factor (rhEGF) concentration was changed to 0.5 ng/mL.

#### BCi-NS1.1 cell line.

Normal airway epithelium-derived basal cell line BCi-NS1.1 cells (18) were cultured in BEGM complete media and maintained in T25 cell culture flasks. For stimulations, cells were seeded in 24-well cell culture plates (1 × 10^5^ cells/well) and used at 80% confluent.

#### ALI cultures.

Primary (p)BECs were obtained from patients with asthma and control subjects bronchoscopically (19). All subjects gave written informed consent, and the study protocol was approved by the Hunter New England Human Research Ethics Committee (05/08/10/3.09). pBECs were grown in BEGM complete media in submerged monolayer culture first and then seeded at 2 × 10^5^ cells in transwells in a 12-well plate (Corning) with ALI-initial media until confluent (at least 3 days in both apical and basal compartments). Once confluent, media was changed to ALI final media by changing rhEGF concentration until day 21 after initial seeding.

#### Patient demographics for pBECs.

Table 1 shows clinical characteristics and inflammatory cell profiles of healthy controls and participants with asthma who were recruited for bronchial brushing collection. Features of participants with asthma were categorized based on clinical severity following GINA guidelines.

**Table 1.** Clinical characteristics and inflammatory cell profiles of healthy controls and participants with asthma who were recruited for bronchial brushing collection

	Healthy	Severe Asthma
Participants, *n*	5	5
Age, yr (median, IQR)	64 (63–64)	53 (50–67)
Sex, *n* (%)	Male 1 (20%), female 4 (80%)	Male 1 (20%), female 4 (80%)
Atopy, *n* (%)	1 (20%)	4 (80%)
FEV1 % predicted, median (IQR)	103 (100–107)	57 (45–61)
FEV1/FVC %, median (IQR)	78.8 (78.47–81.38)	49.5 (46.3–60)
ICS dose, µg budesonide dose/day, median (IQR)	0	2,000 (1,600–2,000)
ACQ5, median (IQR)	NA	3.5 (2.75–4.25)
Neutrophils %, median (IQR)	21.75 (3–25.75)	63.1 (44.2–73.8)
Eosinophils %, median (IQR)	0.25 (0–1)	5.5 (1.25–13.25)
Macrophages %, median (IQR)	38.25 (36.5–39.75)	12.75 (9–27.25)

Values are median (IQR). Features of participants with asthma were categorized based on clinical severity following GINA guidelines. %, percentage; ACQ, asthma control questionnaire; FEV1, forced expiratory volume in 1 s; FVC, forced vital capacity; ICS, inhaled corticosteroids; IQR, interquartile range; *n*, number; yr, years.

#### Bronchial biopsy sample collection and analysis.

cDNA from endobronchial biopsies collected for our previous study that elucidated evidence of ER stress in asthmatic airways was used for correlation analysis. For patient clinical parameters and sample collection details, see Pathinayake et al. (20).

#### Cell culture treatments.

ER stress was chemically induced in cell cultures by adding tunicamycin (2 µg/mL; Merk Millipore) or thapsigargin (1 µM; Merk Millipore) into BEGM minimal media for the indicated time points. A total of 10 µg/mL polyinosinic:polycytidylic acid (poly I:C) was used to induce TSLP induction in BECs.

#### Gene knockdown.

Small interfering (si)RNAs were used for gene silencing. XBP1-silencer siRNA (200 nM, sc-38627; Santa Cruz Biotechnology), CHOP-stealth RNAi siRNA (100 nM, VHS40605; Life Technologies, Australia), IRE1α-silencer-select siRNA (10 nM, s200430; Life Technologies, Australia), ATF6-silencer-select siRNA (10 nM, s223543; Life Technologies, Australia), PERK-silencer-select siRNA (10 nM, s18102; Life Technologies, Australia), and CEBPB silencer-select siRNA (10 nM, s2891; Life Technologies, Australia) with lipofectamine 3000 (ThermoFisher Scientific) were used to knockdown mRNA expression. At 12 h post transfection, media was replenished and at 24 h post transfection cells were treated with interventions.

#### mRNA extraction and qPCR.

Total mRNA was extracted from cells lysed in 350 µL of RLT buffer using RNeasy mini kit (Qiagen, Germany) following the manufacturer’s instructions and quantified using a nano-drop 2000 spectrophotometer (ThermoFisher Scientific). A total of 200 ng of mRNA from each sample was used to produce cDNA using a high-capacity cDNA reverse transcription kit (Applied Biosystems). Various genes of interest were quantified by qPCR using TaqMan gene expression assays (ThermoFisher Scientific, Australia) and normalized to the 18S housekeeping gene. Results were calculated using 2^–ΔΔCt^ (where Ct is the threshold cycle) relative to the mean ΔCt of the healthy control group as described before (21).

#### ELISA.

TSLP protein in cell culture supernatants was measured using a high-sensitivity (3.3–800 pg/mL) human TSLP ELISA kit (ab155444) following the manufacturer’s instructions. Briefly, standards and samples were added into wells and incubated (2.5 h) to bind TSLP to the immobilized antibody. Wells were washed and biotinylated anti-human TSLP antibody was added. After washing away the unbound biotinylated antibody, horseradish peroxidase (HRP)-conjugated streptavidin was added. Wells were again washed, and TMB substrate solution was added. Stop solution was added to stop color development and intensity was measured (450 nm).

### Statistical Analysis

Data were analyzed using GraphPad Prism 9.0.0 software. Multiple comparisons between groups were analyzed with an appropriate test for normally distributed data with ANOVA, followed by post hoc analysis (Dunnett’s or Dunn’s test). Two-group comparisons were analyzed with an unpaired *t* test. Correlation analyses were performed using Spearman’s rank test. Statistical significance of *P* < 0.05 was accepted with a 95% confidence interval.

## RESULTS

### TSLP Gene Expression Is Upregulated in BECs by Chemically Induced ER Stress

**Figure 1.**
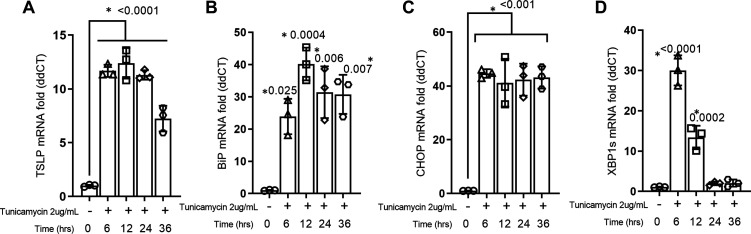
ER stress induces TSLP mRNA expression in bronchial epithelial cells. BCi-NS1.1 cells (*n* = 3) were treated with tunicamycin 2 µg/mL to induce ER stress and UPR markers, and TSLP gene expression was measured by qPCR. ER stress upregulates TSLP (*A*), BiP (*B*), CHOP (*C*), and XBP1s (*D*) gene expression in a time-dependent manner. Differences between the groups were assessed using one-way analysis of variance (ANOVA), with Dunnett’s multiple comparison test. *P ≤ 0.05. ER, endoplasmic reticulum; qPCR, quantitative PCR; TSLP, thymic stromal lymphopoietin; UPR, unfolded protein response.

To examine if ER stress can induce epithelial TSLP expression, BCi-NS1.1 cells were treated with tunicamycin and the mRNA expression of TSLP and key ER stress/UPR markers (BiP, XBP1s, CHOP) were measured time-dependently by qPCR (Fig. 1). Tunicamycin significantly upregulated mRNA expression of TSLP by 6 h post treatment that continued to be elevated until 24 h and at 36 h the mRNA fold change declined (Fig. 1*A*). It also increased each of the key ER stress and the UPR-related markers as early as 6 h post treatment. mRNA of BiP and CHOP mRNA were constantly expressed until 36 h (Fig. 1, *B* and *C*). mRNA expression of XBP1s peaked at 6 h and gradually decreased by 24 h (Fig. 1*D*).

### PERK Signaling in the UPR Is Important for ER Stress-Induced Epithelial TSLP mRNA Expression

**Figure 2.**
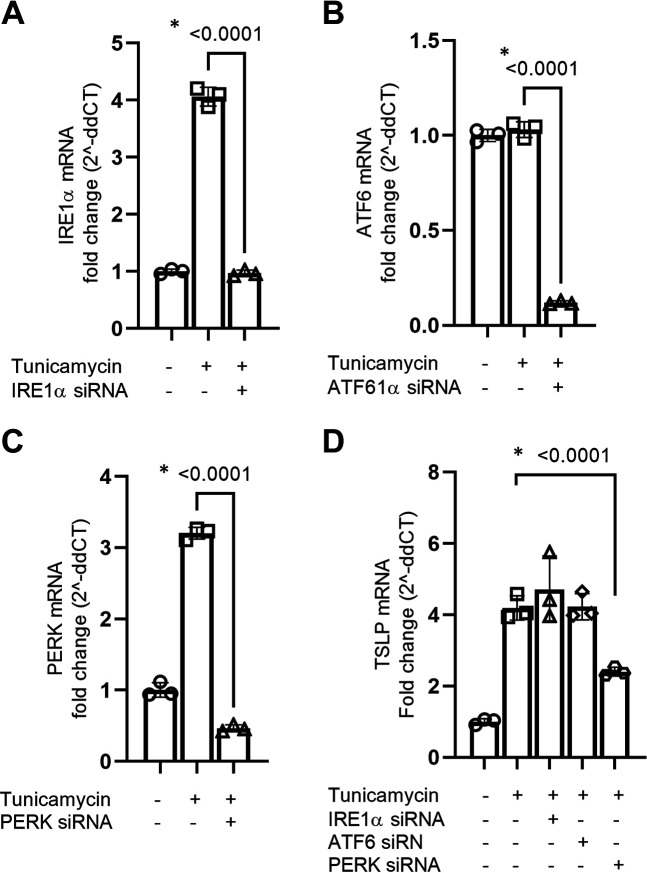
ER stress-induced TSLP mRNA expression is mainly regulated through the PERK signaling pathway. The mRNA expression of IRE1α, ATF6, and PERK in BCi-NS 1.1 cells was knocked down using target-specific silencer-select siRNA (*n* = 3). At 36 h post knockdown, cells were challenged with tunicamycin for 12 h and mRNA expression of IRE1α (*A*), ATF6 (*B*), and PERK (*C*) was measured by qPCR. *D*: mRNA expression of TSLP was measured by qPCR in IRE1α, ATF6, and PERK knockdown cells following tunicamycin challenge. Differences between the groups were assessed using one-way analysis of variance (ANOVA), with Dunnett’s multiple comparison test. *P ≤ 0.05. ER, endoplasmic reticulum; qPCR, quantitative PCR; TSLP, thymic stromal lymphopoietin.

To define which signaling arm of the UPR is important for ER stress-induced TSLP induction, we selectively knocked down the three main branches of the UPR—IRE1a, PERK, and ATF6—using specific siRNAs. After 36 h of siRNA transfection, cells were challenged with tunicamycin for 12 h and harvested for qPCR. All siRNAs significantly knocked down their targets (Fig. 2, *A*–*C*). Notably, only PERK knockdown affected tunicamycin-induced TSLP mRNA induction (Fig. 2*D*) suggesting that ER stress-induced TSLP mRNA expression is regulated through PERK signaling.

### ER Stress-Induced CHOP Expression Is Crucial for Epithelial TSLP mRNA Induction in ER Stress

**Figure 3.**
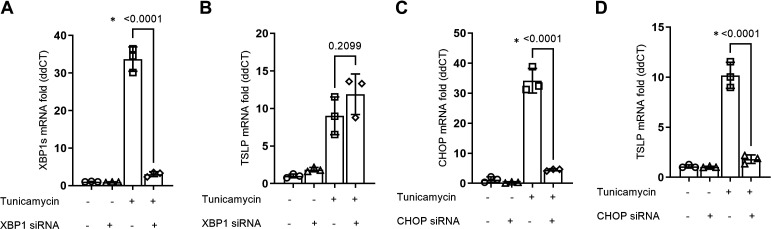
CHOP expression is crucial for the induction of TSLP mRNA expression in ER stress. The mRNA expression of XBP1 and CHOP was knocked down using target-specific silencer-select siRNA (*n* = 3). At 36 h post knockdown, cells were challenged with tunicamycin for 12 h and mRNA expression of XBP1 (*A*), CHOP (*C*), and TSLP (*B* and *D*) was measured by qPCR. Differences between the groups were assessed using Student’s *t* test. *P ≤ 0.05. ER, endoplasmic reticulum; TSLP, thymic stromal lymphopoietin.

Since XBP1s and CHOP are important transcription factors in UPR signaling, and as we have observed that ER stress-induced TSLP gene induction is mainly regulated through the PERK arm of the UPR, we examined if these transcription factors are involved in TSLP expression in airway epithelial cells. To demonstrate, we knocked down XBP1s and CHOP expression with siRNA and the expression of TSLP with or without tunicamycin challenge was measured by qPCR. XBP1s knockdown (Fig. 3*A*) did not have any significant effect (Fig. 3*B*). Knockdown of CHOP (Fig. 3*C*) significantly reduced tunicamycin-induced TSLP mRNA expression (Fig. 3*D*). Collectively, these results show that the PERK-CHOP signaling axis is crucial for ER stress-induced epithelial TSLP gene induction.

### CHOP Is a Transcription Enhancer for AP-1 to Induce TSLP Expression in the Airway Epithelium

**Figure 4.**
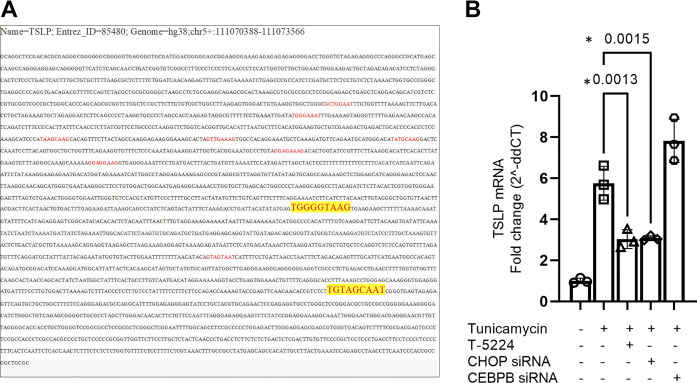
CHOP-AP1 complex is crucial for ER stress-induced TSLP gene induction in the airway epithelium. *A*: consensus recognition sequences for CHOP ( ATTGCATCAT) and CEBPB (T[TG]NNGNAA[TG]) were screened within the complete nucleotide sequence of TSLP. *B*: mRNA expression of CEBPB was knocked down using siRNA, and AP-1 expression was inhibited using T-5224 (*n* = 3). Cells were then treated with tunicamycin and the mRNA expression of TSLP was measured by qPCR. Differences between the groups were assessed using one-way analysis of variance (ANOVA), with Dunnett’s multiple comparison test. *P ≤ 0.05. ER, endoplasmic reticulum; qPCR, quantitative PCR; TSLP, thymic stromal lymphopoietin.

Since our data show that CHOP is crucial for ER stress-induced TSLP mRNA expression in BECs, we assessed if CHOP can act as a transcription factor for TSLP induction. We first screened the complete nucleotide sequence of TSLP for matches for the consensus recognition sequence for CHOP. However, we did not find any matching consensus sequence suggesting that CHOP cannot directly act as a transcription factor for TSLP (Fig. 4*A*). CHOP cannot form homodimers but forms stable heterodimers with C/EBP family proteins and is known to act as a transcription factor (22). CHOP also forms a heterodimer with CCAAT enhancer binding protein β (CEBPB) and components of the AP-1 protein. Thus, we assessed the consensus recognition sites for CEBPB and found them in the TSLP nucleotide sequence. AP-1 is a known regulator of TSLP transcription (23), can form a heterodimer with CHOP, and activates the promoters of selected genes such as somatostatin, JunD, and collagenase (16). Using specific siRNA knockdown of the CEBPB gene and an AP-1**-**specific inhibitor T-5224, we determined their role in ER stress-induced TSLP gene transcription (Fig. 4*B*). AP-1 inhibition significantly reduced tunicamycin-induced TSLP mRNA expression, comparable to the effect of CHOP siRNA knockdown. Knockdown of CEBPB did not affect tunicamycin-induced TSLP mRNA expression. These results show that the CHOP-AP-1 complex, but not CEBPB, is crucial for ER stress-induced TSLP gene transcription.

### ER Stress Alone Does Not Induce TSLP Protein in BECs but Synergically Enhances Its Production by Poly I:C

**Figure 5.**
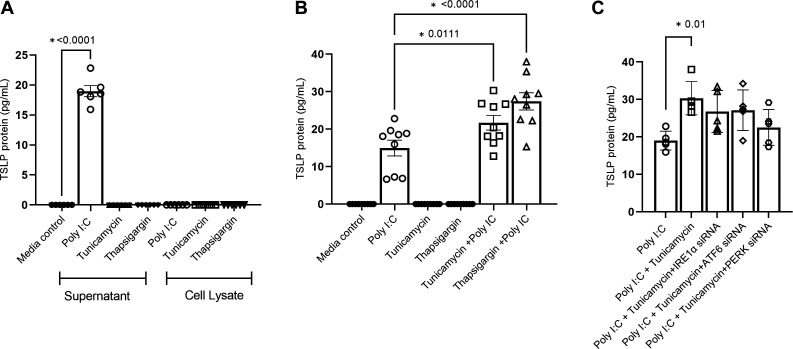
ER stress alone does not induce but synergically increases the production of TSLP protein via TLR3 signaling. *A*: BCi-NS1.1 cells were challenged with various ER stress inducers and poly:IC as a positive control (*n* = 6). After 48 h post challenge, TSLP protein levels in cell supernatants and lysates were measured by ELISA. *B*: BCi-NS1.1 cells were challenged with various ER stress inducers with or without poly:IC (*n* = 9) and after 48 h the TSLP protein level in the supernatant was measured by ELISA. *C*: to determine the effect of UPR signaling arms on poly IC-induced TSLP protein expression, IRE1α, ATF6, and PERK genes were knocked down using target-specific silencer-select siRNA in BCi-NS1.1 cells (*n* = 5) and treated with poly IC together with tunicamycin. After 48 h, secreted TSLP was measured by ELISA. Differences between the groups were assessed using one-way analysis of variance (ANOVA), with Dunnett’s multiple comparison test. *P ≤ 0.05. ER, endoplasmic reticulum; TSLP, thymic stromal lymphopoietin.

Our mRNA expression data showed significant induction of TSLP expression with chemical-induced ER stress in BECs. We next examined if this translated into protein. To do this, we measured secreted TSLP protein in cell culture supernatants of BCi-NS1.1 cells challenged with tunicamycin or thapsigargin. Intriguingly, we did not find any detectable levels of TSLP in the culture supernatants (Fig. 5*A*). As ER stress activates signaling pathways that inhibit protein export and secretion, we measured TSLP protein in cell lysate by ELISA to determine if TSLP protein is translated but then trapped inside the cells. However, we could not detect any TSLP protein in the whole cell lysate even using a high-sensitivity ELISA kit. To assess if ER stress-induced TSLP mRNA induction can enhance the TSLP protein secretion by other known TSLP stimulants in the airway epithelium, we treated BCi-NS1.1 cells with poly I:C alone or together with tunicamycin or thapsigargin for 48 h and the secreted TSLP protein level was measured by ELISA. After 48 h of challenge, poly I:C together with tunicamycin or thapsigargin resulted in the production of significantly higher levels of TSLP protein compared with poly I:C alone (Fig. 5*B*). To determine if knockdown of UPR signaling arms affects poly IC + tunicamycin**-**induced TSLP protein production, the gene expression of PERK, IRE1α, and ATF6 in BCi-NS 1.1 cells was knocked down using siRNA. Cells were then treated with poly IC along with tunicamycin and after 48 h secreted TSLP was measured by ELISA (Fig. 5*C*). Our data show that knockdown of PERK gene slightly reduced poly IC + tunicamycin**-**induced TSLP protein but not statistically significant.

### ER Stress Induces TSLP mRNA Expression in Differentiated pBECs and Clinically Correlates with Severe Neutrophilic Asthma

**Figure 6.**
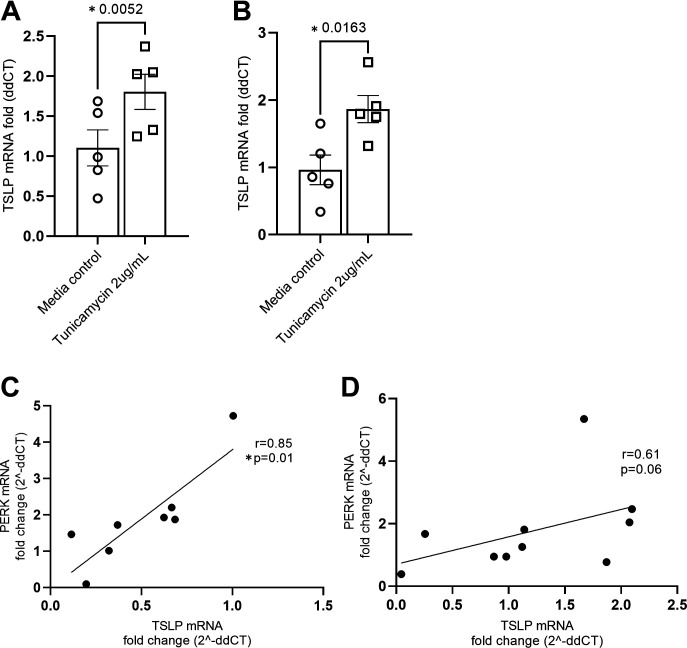
ER stress induces TSLP in primary bronchial epithelial cells (pBECs) and correlates with TSLP expression in severe neutrophilic asthma. pBECs-ALI cultures from control subjects (*n* = 5; *A*) and patients with asthma (*n* = 5; *B*) were challenged with tunicamycin, and TSLP mRNA induction was measured by qPCR. The association between PERK and TSLP and mRNA expression in endobronchial biopsy samples from patients with neutrophilic (*n* = 8; *C*) and eosinophilic asthma (*n* = 10; *D*) was assessed by Spearman’s correlation analysis. *P ≤ 0.05. ER, endoplasmic reticulum; qPCR, quantitative PCR; TSLP, thymic stromal lymphopoietin.

Single-cell-transcriptomic data from lung atlas data suggest that airway epithelial basal cells are the predominant source of TSLP production (24). This determined our choice of BCi-NS 1.1 minimally immortalized bronchial epithelial basal cell line, grown in submerged culture to reflect this basal cell phenotype. To validate our findings, we treated pBEC-ALI cultures from asthma patients and control subjects with ER stress inducers (patients’ demographic details are displayed in Table 1). Similar to our basal cell line data, pBECs from both control subjects (Fig. 6*A*) and patients with severe asthma (Fig. 6*B*) (*n* = 5) had a significant increase in TSLP mRNA induction upon tunicamycin challenge. To assess if increased ER stress is associated with increased expression of TSLP in clinical samples, a correlation analysis was performed measuring the mRNA expression of PERK and TSLP in bronchial biopsies from patients with severe neutrophilic (*n* = 8) and eosinophilic asthma (*n* = 10) (patient’s demographic details are displayed in Table 2). Increased ER stress and TSLP mRNA positively and significantly correlate in severe neutrophilic asthma (Fig. 6*C*) but did not reach significance in those with severe eosinophilic asthma (Fig. 6*D*).

**Table 2.** Clinical characteristics and inflammatory cell profiles of healthy controls and severe asthma patients in whom endobronchial biopsies were collected

	Neutrophilic Asthma	Eosinophilic Asthma
Subjects, *n*	8	10
Age, yr, mean (SD)	61.9 (10.94)	56.6 (9.58)
Males/females, *n*	4/4	4/6
Atopy, %	70	70
FEV1 % predicted, median (SD)	66.5 (18.79)	74.5 (17.5)
FEV1/FVC%, median (SD)	62 (6.2)	67 (7.33)
ICS dose, as µg budesonide dose/day, median (IQR)	1,000 (1,000–1,000)	900 (700–1,000)
ACQ average, median (IQR)	2.3(1.765–2.65)	2.3(1.55–2.815)
Neutrophils %, median (IQR)	88.51 (85.25–91.88)	24.27 (15.06–26)
Eosinophils %, median (IQR)	1.05 (0.56–1.62)	29.57 (14.4–42.5)
Macrophages %, median (IQR)	8.9 (5.87–12.06)	31.5 (21.3–39.31)

Values are median (IQR). Features of severe asthma participants were further categorized based on inflammatory phenotypes. %, percentage; ACQ, asthma control questionnaire; FEV1, forced expiratory volume in 1 s; FVC, forced vital capacity; ICS, inhaled corticosteroids; IQR, interquartile range; *n*, number; yr, years.

## DISCUSSION

TSLP is an epithelial-derived cytokine that initiates potent inflammatory responses implicated in the pathogenesis of severe asthma (25, 26). Recent findings in clinical trials with anti-TSLP monoclonal antibody (tezepelumab) in uncontrolled severe asthma patients showed a significant reduction in asthma exacerbations and improved symptoms, an effect that was not confined to participants with eosinophilic airway inflammation (27). This suggests that there is a dominant role for TSLP in corticosteroid-refractory severe asthma. Other clinical benefits were associated with reduced levels of a broad spectrum of Th2 cytokines, and baseline biomarkers (e.g., blood eosinophils, IgE, FeNO) were observed across a range of severe asthma phenotypes (28). This suggests that anti-TSLP elicits broad inhibitory effects on pathways that are key to inflammation in asthma rather than on narrower inhibition of individual downstream factors. The airway epithelium in asthma is highly modified, fragile, and activated (26). Chronic inflammation, persistent cellular stress, and the fragile nature of the asthmatic epithelium result in constant damage and repair that lead to high metabolic turnover (29–31). These factors activate the UPR and some pathways of the integrated stress response. We and others previously showed that ER stress and the UPR are associated with severe corticosteroid-resistant neutrophilic and eosinophilic asthma (12, 32–34). We have also shown robust evidence of altered ER stress and the UPR signaling in an array of clinical samples of asthma and its correlation with disease severity and inflammatory phenotypes (20). Importantly, we found that these ER stress and the UPR markers are more confined to metabolically active cells with greater turnover such as the airway epithelium in asthmatic lung biopsies (20).

Our findings in the current study demonstrate that ER stress-induced activated UPR signaling contributes to TSLP mRNA induction in BECs and enhances the TSLP secretion induced by TLR3 agonists. This could be a novel mechanism that explains upregulated TSLP secretion in the asthmatic airways and consequent asthma exacerbations. The underlying ER stress in the asthmatic airway epithelium may then enhance TSLP secretion induced by respiratory viruses and allergens that trigger TLR3 signaling. Indeed, it has been shown that rhinovirus infection in the asthmatic airways exaggerates TSLP secretion compared with normal airways (3).

In this study, interestingly, we found that ER stress alone significantly induces TSLP gene expression but not protein release. Protein translation repression mechanisms induced by ER stress via eIF2α phosphorylation may suppress the translation of TSLP mRNA induced by ER stress. However, this process was not affected or was overcome when ER stress occurred along with TLR3 activation. Under ER stress conditions, extensive remodeling in translatomes occurs and translation of nearly 50% of mRNA is affected; however, certain mRNAs are resistant to this translational repression (35). Some transcription factors including subunits of AP-1 (Jun and Fos) increase under ER stress (35). Whether PERK-CHOP-induced TSLP mRNA undergoes this eIF2α-initiated translational repression is unknown. Potentially, TLR3-induced TSLP mRNA is resistant to this process and enhanced via transcription factor upregulation during ER stress. Furthermore, ER stress has been shown to induce NF-κB activity and enhance TLR signaling (36, 37). Further studies to elucidate the mechanisms underpinning ER stress upregulation of TLR3-induced TSLP would be required.

ER stress also enhances *C. albicans-* and β‐glucan-induced TSLP secretion in DCs. One study demonstrated that fungus-induced TSLP requires an integration of signals from dectin‐1, the IL‐1 receptor, and ER stress signaling pathway particularly IRE1-α and PERK though not CHOP (11). In contrast, our study shows that the PERK-CHOP signaling pathway is important in ER stress-induced TSLP gene induction in airway epithelial cells, though it requires priming signals such as TLR3 activation to produce and release TSLP protein. In consistent with this, knockdown of PERK signaling arm marginally reduced the poly IC + tunicamycin-induced TSLP protein secretion (Fig. 5*C*) but was not statistically significant. This could be due to the CHOP gene expression, which is the main factor involved in ERS-induced TSLP, and could still be activated via multiple ERS arms although mainly regulated through PERK signaling (38). Moreover, TLR3-induced TSLP production in airway epithelial cells is also shown to be induced in an IRF3-NF-κB-dependent manner and synergically enhanced by Th2 cytokines such as IL-4 and IL-13 explaining higher TSLP secretion in asthmatic airways (39). Whether ERS enhances IRF3-NF-κB and contributes to enhance the TLR3-induced TSLP protein is unknown and needs future investigation.

We previously showed evidence of increased ER stress and UPR markers in the airways of asthmatic patients, particularly in severe eosinophilic and neutrophilic asthma (20). We found these upregulated markers in BALF, sputum, and endobronchial biopsies taken from severe asthma patients. In this study, we show that induced ER stress, via the PERK signaling pathway, correlates with increased TSLP gene expression in endobronchial biopsy samples of patients with severe neutrophilic asthma, where we mostly find severe refractory disease (40). Upregulated and persistent ER stress in neutrophilic asthma airways may prime TSLP that is then enhanced further upon TLR3 activation such as by respiratory virus infections and contributes to severe exacerbations, leading to further neutrophilic airway inflammation and a state that is relatively refractory to corticosteroid treatment (41). Treatments that suppress ER stress in the airways such as pharmacological chaperones may provide novel therapeutic avenues to reduce TSLP release, making them a relatively low-cost potential treatment for severe asthma or potentially in the setting of acute asthma exacerbations triggered by virus infections, in uncontrolled asthma.

## DATA AVAILABILITY

The data that support the findings of this study are openly available at https://doi.org/10.6084/m9.figshare.24047154.

## GRANTS

This work was funded by the National Health and Medical Research Council (NHMRC) of Australia (APP2002953). P.M.H. is funded by Fellowship/Investigator grants from the NHMRC (1175134) and University of Technology Sydney.

## DISCLOSURES

No conflicts of interest, financial or otherwise, are declared by the authors.

## AUTHOR CONTRIBUTIONS

P.S.P., A.C.-Y.H., and P.A.B.W. conceived and designed research; P.S.P. and K.S.N. performed experiments; P.S.P. and K.S.N. analyzed data; P.S.P., A.C.-Y.H., J.C.H., and P.A.B.W. interpreted results of experiments; P.S.P. prepared figures; P.S.P. and A.C.-Y.H. drafted manuscript; P.S.P., A.C.-Y.H., K.S.N., J.C.H., P.M.H., and P.A.B.W. edited and revised manuscript; J.C.H., P.M.H., and P.A.B.W. approved final version of manuscript.
